# Advancing stone treatment: the transformative impact of flexible navigable suction ureteral access sheath (fans) for inferior calyx calculi (<2 cm)

**DOI:** 10.3389/fruro.2025.1695781

**Published:** 2026-01-23

**Authors:** Xiang yun Xu, Hong jian Tu, Qing hua Luo, Wen Wei, Fan qi Cheng, Lei hua Cao

**Affiliations:** 1 Department of Urology, Nanchang People's Hospital, Nanchang, Jiangxi, China; 2 Machu Health Center, Nanchang, Jiangxi, China

**Keywords:** urolithiasis, flexible navigable suction ureteral access sheath, Inferior calyx calculi, RIRS, stone-free rates

## Abstract

**Objective:**

This study aimed to assess the efficacy, safety, and stone-free rates (SFR) of the application of a flexible navigable suction ureteral access sheath (FANS) in patients undergoing retrograde intrarenal surgery (RIRS) for inferior calyx calculi (<2 cm).

**Methods:**

A retrospective analysis of the clinical data of 111 patients who underwent treatment for inferior calyx calculi at Nanchang People’s Hospital between January 2023 and October 2024 was performed. Patients undergoing RIRS with laser lithotripsy were evenly distributed into two groups: FANS with continuous flow lithotripsy in ureterorenolithotripsy procedures (F-Group), a conventional sheath in ureterorenolithotripsy procedures (C-Group).

**Results:**

A significantly greater stone clearance rate (96.23% vs. 81.03%, p=0.017) and shorter operative time (37.81 ± 6.07 min vs. 46.59 ± 6.36 min, p<0.001) were observed with the F-Group. Basic attributes, including demographics of patients and stone properties, the infundibulo-pelvic angle (IPA), length of hospital stay, and complications, were compared between the groups and found to be approximately the same.

Conclusions

FANS significantly improves SFR without increasing postoperative complications or negatively affecting recovery, offering a promising alternative to conventional sheaths in ureterorenolithotripsy procedures for inferior calyx calculi. FANS improves kidney stone surgery outcomes, is feasible and safe for inferior calyx calculi (<2 cm).

## Introduction

Urinary stones are most commonly found in the kidney, accounting for approximately 40% to 50% of cases. Moreover, inferior calyx calculi account for approximately 36% of all kidney stones. Owing to gravity, the lower part of the kidney, known as the inferior calyx, collects toxins, crystals, and other substances excreted from urine. This accumulation leads to the formation of stones, which are difficult to discharge because of their low position. There are three main treatment options for an inferior calyx calculus with a diameter less than or equal to 2 cm. Relevant studies have indicated that extracorporeal shock wave lithography (ESWL), percutaneous nephrolithotomy (PCNL), and retrograde intrarenal surgery (RIRS) are effective treatment options for inferior calyx calculi measuring 1~2 cm. The stone removal rate ranges from 52% to 67.7% for ESWL, with potential risks including secondary or multiple stone removal and the formation of “stone streets” and perirenal hematomas (1–4). PCNL requires ultrasound guidance, is relatively invasive, and is associated with various complications, such as intraoperative and postoperative bleeding and infection, with rates as high as 50% (5). In 2017, RIRS was recommended as the first-line treatment for inferior calyx calculi less than 2 cm in size in the European Guidelines for the Diagnosis and Treatment of Urinary Calculi (6). Compared with PCNL, RIRS allows entry into the renal pelvis and removal of calculi through the natural cavity. It is considered a relatively noninvasive procedure, and patients are more willing to accept it.

RIRS approach has been demonstrated to be both safe and effective for managing renal calculi of this size (6). Traditionally, the procedure involves the use of RIRS in conjunction with a ureteral access sheath(UAS). UAS are integral to Retrograde Intrarenal Surgery (RIRS) by enhancing surgical visibility, reducing intrarenal pressure (IRP), and minimizing the risk of postoperative infectious complications (7–9). However, traditional ureteral access sheaths are limited by their rigid distal ends and lack of negative pressure suction capabilities, which often hinder access to the inferior calyces of the kidney. A recent advancement in this domain is the flexible navigable suction ureteral access sheath (FANS), which has been developed to improve stone clearance during RIRS (10). FANS offers continuous suction, theoretically decreasing operative time and the necessity for laser ablation, thereby enhancing the stone-free rate (SFR) by preventing stone fragments from obstructing the operative field (11). The FANS incorporates a flexible tip that moves in conjunction with the ureteroscope and an integrated suction mechanism that actively removes irrigation fluid, stone fragments, and debris. The F-Group utilizes a flexible ureteroscope in combination with FANS, which provides improved access to the inferior calyces and facilitates the aspiration of stone fragments under direct visualization. The safety, efficacy, and potential advantages of this latter approach were assessed by comparing clinical data, including operation time and SFR, between the two groups. This research aims to provide essential insights into improving treatment strategies for this particular group of patients by examining these minimally invasive methods.

## Materials and methods

This study was approved by the Ethics Committee of Nanchang People’s Hospital (K-ky2022045). All patients provided written informed consent. Patients who underwent FURS under general anesthesia in the Department of Urology between January 2023 and October 2024 and who were diagnosed with a single lower pole renal stone <2 cm were included. This retrospective cohort study included 111 patients who had inferior calyx stones treated with RIRS using a Holmium: YAG laser. Patients who were also treated for other simultaneous stones were not included.

–C-Group, consisting of patients treated with a conventional standard UAS.
–F-Group, consisting of patients treated with the FANS.

### Inclusion and exclusion criteria

The inclusion criteria included patients with a single stone measuring 1–2 cm and those aged over 18 years. Patients were excluded if they had multiple stones, stones that were very small or large, were under 18 years old, had a BMI over 40 kg/m², or had a history of bleeding disorders, renal anatomical issues, distal obstructions, or musculoskeletal deformities.

### Surgical techniques

**Figure 1:**
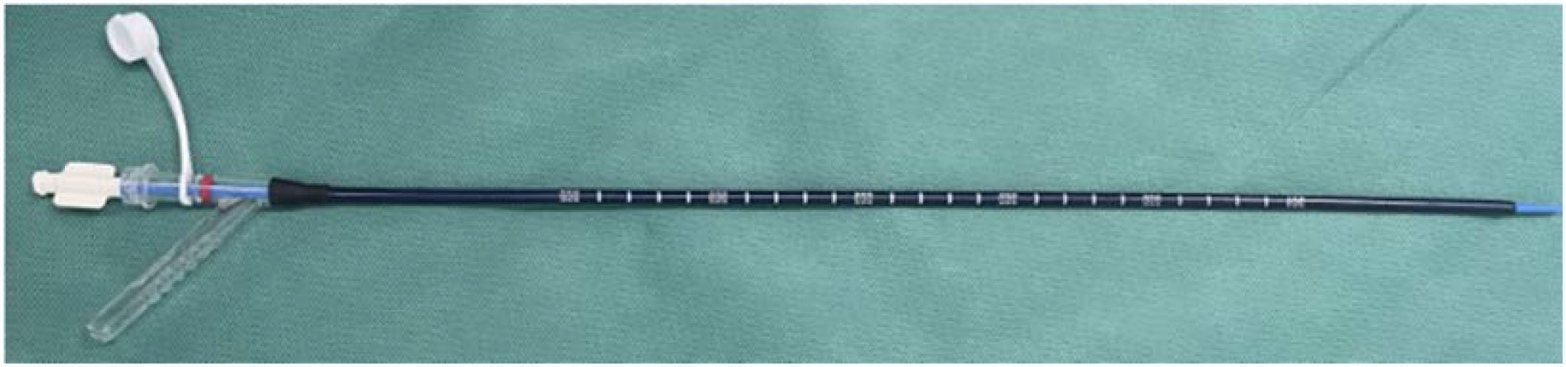
Flexible navigable suction ureteral access sheath (FANS) .

**Figure 2:**
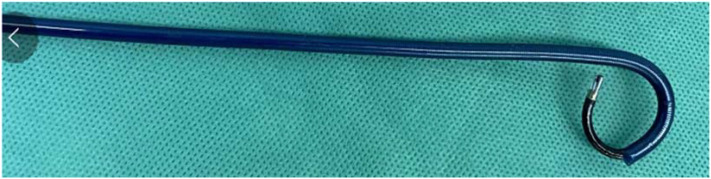
Bending angle of the flexible ureteroscope guided FANS.

**Figure 3:**
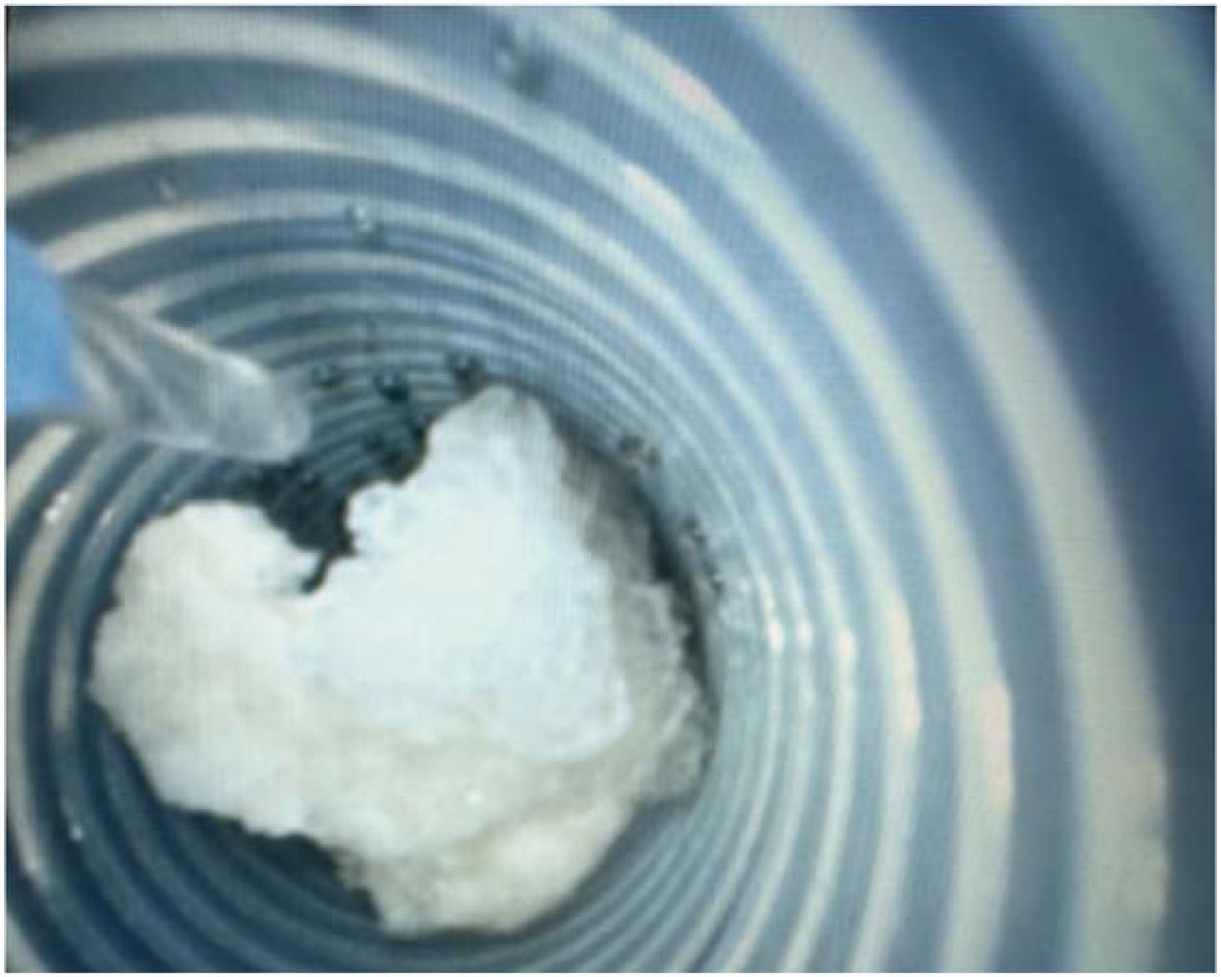
Stone fragments are suctioned out through the FANS.

C-Group: Intravenous anesthetics were administered, the perineal area was disinfected, lithotomy was performed, and a guide-wire was placed up to the upper ureter via a WOLF (6/8 Fr) ureteroscope. To reach the kidney, a ureteral access sheath (the length was 45 cm for male patients and 35 cm for female patients, a 10–12 Fr, Pulin, Guangzhou, China) was inserted, and the REDPINE digital flexible ureteroscope was passed through the working channel. An irrigation pressure of 100–200 ml/min was used (STORZE). The flexible ureteroscope was bent into the inferior calyx to locate the stone. The stones were disintegrated using a Lumenis Holmium: YAG laser with 200 μm fibers, set to 1–1.5 J and 30–40 Hz. The stone was fragmented as thoroughly as needed, and a retrieval basket was used to collect any sizable pieces. The renal pelvis and all calyces were inspected for debris, and a double -J (5–6F/28 cm)stent was inserted at the conclusion. F-Group: The procedure for placing the guide wire was the same as that described above. A FANS 10–12F (the length was 45 cm for male patients and 35 cm for female patients, a 10–12 Fr, Hua Mei Medical YWF, GuangDong, China) (**Figure 1**) was inserted to reach the kidney, and a REDPINE digital flexible ureteroscope was inserted via the working channel. An irrigation pressure of 100–200 ml/min was used (STORZE). The flexible ureteroscope was used to drive the front-end bendable negative pressure ureteral access sheath flexure together into the inferior calyx (**Figure 2**). A Lumenis Holmium: YAG laser equipped with 200 μm fibers and configured to 1.5–2 J and 20 Hz was employed to break down the stones. Suitable stone fragments (2–3 mm) were suctioned out of the body one by one to adjust the negative pressure (**Figure 3**). Debris was inspected in the renal pelvis and calyces, and a double J was positioned at the end.

### Criteria and statistic

Preoperative assessment involves gathering a comprehensive medical history, conducting a physical exam, performing a urine culture, evaluating renal function, doing blood tests, and obtaining radiological images: KUB (Kidney-ureter-bladder radiography), IVU (Intravenous urography) and non-contrast CT (non-contrast Computed Tomography) abdomen/pelvis. Document and compute the Body Mass Index (BMI) in kilograms per square meter (kg/m²), stone laterality, stone length in millimeters (mm), stone dimensions in terms of width (mm), stone density measured in Hounsfield Units (HU), and the Infundibulopelvic Angle (IPA°). Prior to the surgical procedure, including throughout the perioperative period, both blood pressure and blood glucose levels were effectively stabilized to meet the preoperative requirements. Additionally, the urinary tract infection was managed, and the patient remained afebrile. KUB was utilized to evaluate any remaining stones. The stone-free rates (SFR) was determined by the lack of clinically significant fragments larger than 2 mm. IVU was used to evaluate IPA. Operative times were recorded from placed a guide wire to place a double J.

The data was analyzed and processed using IBM SPSS Statistics version 26 software. A Q-Q plot was used to test all numeric variables for normality. All continuous variables that followed a normal distribution were displayed as mean ± standard deviation and assessed using the independent t test. The Mann-Whitney U test was used to analyze variables that did not follow a normal distribution. The counting data were represented as percentages and compared using the χ2 test. Fisher’s exact test was used when expected counts were less than 5. All p-values were two-tailed, with a significance threshold of less than 0.05.

## Results

Firstly, details of patients and stones were presented in **Table 1**. There were no statistically significant differences between the C-Group and the F-Group regarding the following parameters: Body Mass Index (BMI, kg/m²), stone laterality, stone size length (mm), stone size width (mm), stone density (Hounsfield Units, HU), and infundibulopelvic angle (IPA°). The study comprised a total of 111 cases. Notably, the stone density predominantly approximated 1000 HU, and the IPA° consistently exceeded 40°. For a comprehensive overview, please consult **Table 1**.

**Table 1:** Details of patients and stones.

Data	F-Group (n=53)	C-Group (n=58)	*P*-value
No. Patients	53	58	-
Patient Age (year)	46.09±15.59	43.93±13.27	0.432
Gender,n(%)			0.842
Male	31(58.49)	35(60.34)	
Female	22(41.51)	23(39.66)	
BMI (kg/m^2^)	22.68±1.18	22.91±1.23	0.324
Stone Laterality,n(%)			0.820
Left	34(64.15)	36(62.07)	
Right	19(35.85)	22(37.93)	
Stone Size Length(mm)	13.84±1.47	13.46±1.27	0.145
Stone Size Wigth(mm)	6.29±0.97	6.60±1.06	0.110
Stone Density (HU)	1033.09±159.33	1016.17±143.13	0.557
IPA°	57.40±7.73	58.14±7.16	0.601

As shown in **Table 2**, an obviously shorter operative time (37.81 ± 6.07 min vs. 46.59 ± 6.36 min, p<0.001) was observed in the F-Group. According to the SFR evaluation, the overall SFR of F-Group improved significantly after surgery (96.23% compared to 81.03%,p=0.0017) and there was still a significant difference two weeks post-surgery (96.23% versus 82.76%, p=0.031). Severe postoperative colic pain, a common and more frequent complication, was gradually relieved with pain medication. There were no significant differences in postoperative complications between the two groups (P>0.05). The duration of the surgical procedure for all 111 cases was maintained within 90 minutes. The SFR at the two-week mark showed an increase in the C-Group, whereas the F-Group exhibited no statistically significant change. Intraoperative complications, such as ureteral injury, ureteral rupture or tearing, and severe bleeding, were absent in both groups. The necessity for re-intervention was minimal, with only 2 patients in the F-Group requiring a second procedure, compared to 10 patients in the conventional sheath group.

**Table 2:** Information on surgery and its results.

Surgical data	F-Group (n=53)	C-Group (n=58)	*P*-value
Operative Time (min)	37.81±6.07	46.59±6.36	<0.001
Postoperative Hospital Stay (day)	2.70±0.70	2.88±0.75	0.191
Postoperative Complication			0.745
no complication	44	45	
fever	4	5	
bleeding	0	1	
sever colic pain	5	7	
Stone-free Rate (one-day)	96.23%(51/53)	81.03%(47/58)	0.017
Stone-free Rate (two-weeks)	96.23%(51/53)	82.76%(48/58)	0.031

## Discussion

As a result of advancements in technology and medical treatment, RIRS has become more efficient and feasible for treating urolithiasis because of improvements in surgeon performance and visualization. These advancements, coupled with the increased demand for minimally invasive procedures, have made the procedure more appealing to patients with urinary calculi (12). In RIRS, there is no need for “dead angle” lithotomy or exploration of more than 95% of the renal assembly system area (13, 14). The new generation of flexible ureteroscope can actively bend up to 280°, and the end of the 6.7~7.2 cm lens can be shifted twice. RIRS has few limitations in treating renal stones, but inferior calyx calculi are the most difficult to treat. The flexible ureteroscope must be actively bent at a large angle so as to enter the inferior calyx to observe the calculi (15). The FANS (length 45 cm for male and 35 cm for female, 10–12 Fr) in this study contains an inner core and an outer sheath. The inner core of the FANS resembles that of an ordinary ureteral access sheath, but the outer sheath is an evolved iteration of the conventional ureteral access sheath. In contrast to that of the traditional sheath, the outer sheath of the FANS has two supplementary functions. Firstly, the flexible tip of the 6-8cm tube is flexible and reinforced with springs in the bending area, which can be guided by the front end of the flexible ureteroscope to bend. Secondly, the distal end oblique lateral side branch of the sheath can be connected to a negative pressure suction container, and the amplitude and frequency of negative pressure suction can be manually adjusted(**Figure 2**).

In comparison to UAS, FANS offers several distinct advantages (16). Primarily, it employs negative pressure aspiration through an oblique lateral side branch of the sheath, enabling the surgeon to adjust and control suction pressure in real-time. This capability ensures optimal suction, thereby preventing both insufficient and excessive aspiration (17), and contributes to maintaining a clearer surgical field. According to the hydrodynamic effect theory and the vacuum cleaner effect in continuous-flow systems, stone fragments situated within 1 cm of the endoscope tip, near or at the sheath opening, are most effectively suctioned towards the gap between the endoscope and the sheath, facilitating their efficient removal (18, 19). Furthermore, the immediate suctioning of debris and dust reduces bacterial presence and limits the absorption of endotoxins, which contributes to a higher SFR, a lower incidence of infectious complications (20), and a diminished need for stone baskets or forceps (21). Our findings underscore the advantages of FANS, particularly its significantly higher SFR at one day postoperatively (96.23% compared to 81.03%, p = .0017), indicating superior efficacy in stone clearance, as previously demonstrated (22, 23). The likelihood of recurrent procedures, which are often costly, time-consuming, and associated with increased patient discomfort, is reduced. These findings are consistent with prior research indicating that FANS technology improves the clearance of stone fragments during procedures, reduces the need for additional fragment extraction, minimizes the necessity for re-intervention without significant complications, and decreases the risk of stone recurrence (24, 25).

**Figure 4:**
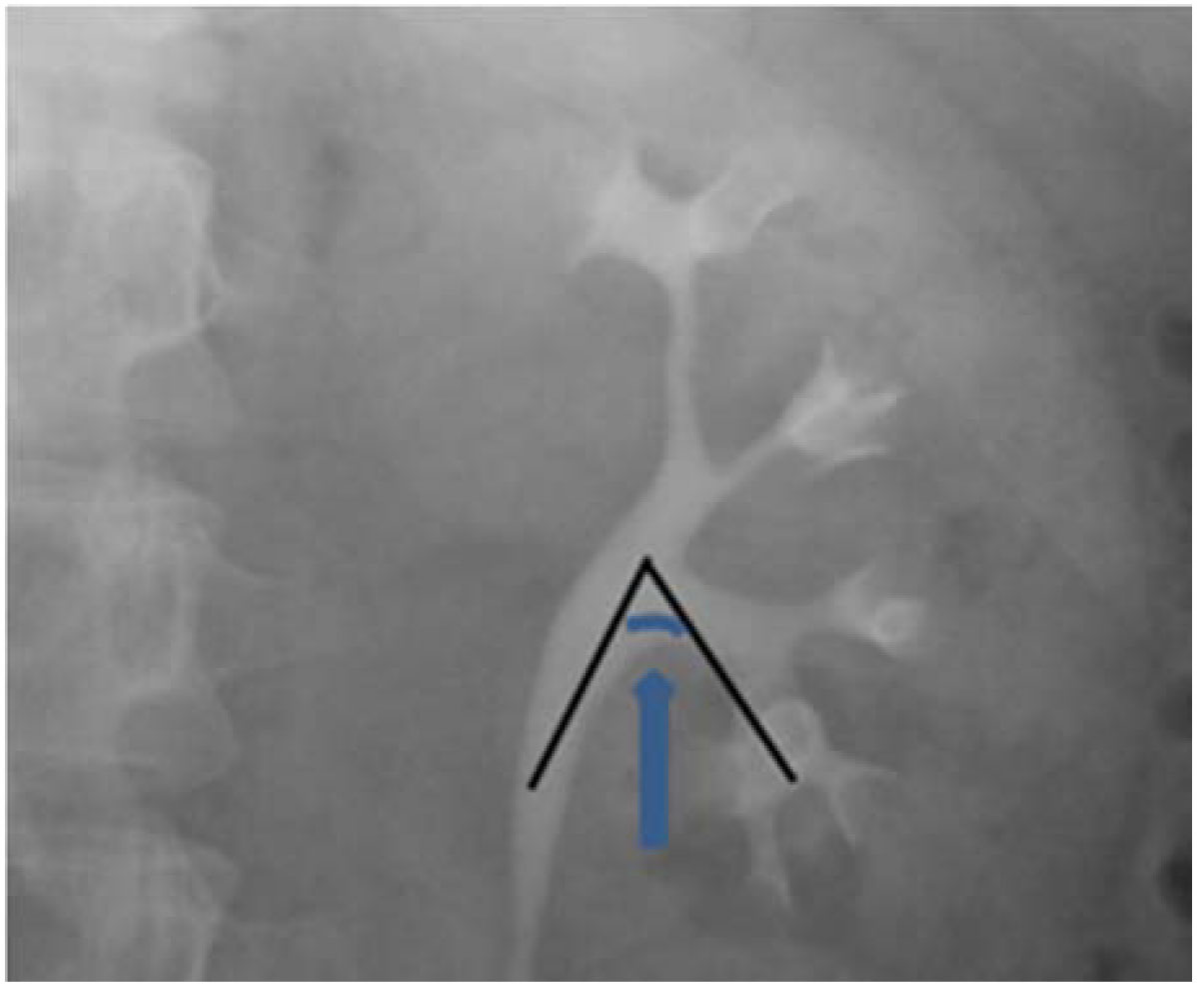
IPA, infundibulo-pelvic angle.

The anatomical configuration of the subcalyx significantly influences its accessibility. The intersection of the central axis of the renal pelvis and the central axis of the inferior calyx, where the stone is situated, is referred to as the infundibulo-pelvic angle (IPA) (26) (**Figure 4**). Current research identifies the IPA as the most critical and extensively studied anatomical factor in this context (26). However, studies have demonstrated that when the IPA exceeds 35°, a flexible ureteroscope can access the inferior calyces (14, 27). Consequently, the initial routine preoperative assessment of patients’ stone IPA values using IVU in this study did not reveal IPA values less than 35 degrees, which was not related to exclusion criteria or sample size. The F-group procedure is characterized by high energy and low frequency (fragmentation mode), facilitating the fragmentation of stones into channels accessible via the ureteral access sheath. The FANS utilizes a flexible ureteroscope to access the inferior calyx. This approach offers excellent operative visualization, allowing for the extraction of stone fragments individually from the body. The procedure can potentially reduce operative time and surgical complexity. Notably, the FANS group demonstrated a significantly shorter operative time compared to the C-group (37.81 ± 6.07 minutes vs 46.59 ± 6.36 minutes, p<0.001). This reduction in operative time may decrease procedural fatigue for the surgeon and lower the risk for the patient associated with prolonged anesthesia exposure or infection during lithotripsy. No significant differences were observed between the two groups regarding intraoperative and postoperative complications, with no serious complications reported overall. These findings suggest that FANS technology may offer transformative improvements in surgical outcomes for inferior calyx calculi less than 2 cm in size.

### Limitations

Our prospective study is subject to certain limitations. Firstly, the sample size was relatively small, which may constrain the generalizability of the findings. Secondly, although FANS could theoretically reduce intrarenal pressure (IRP), our study did not include a pressure feedback device for measuring real-time IRP, necessitating further validation. Despite these limitations, the results of this study provide compelling evidence supporting the use of FANS in enhancing surgical outcomes, such as reducing operative time and re-intervention rates, in RIRS procedures. To confirm these findings, larger, multicenter studies with extended follow-up periods are required.

### Conclusions

The findings from this study suggest that the FANS offers significant advantages over conventional sheaths in RIRS procedures for inferior calyx calculi (<2 cm). The FANS resulted in a higher SFR and shorter operative time, without increasing postoperative complications or negatively affecting recovery. FANS appears to be a promising tool in enhancing the efficiency and outcomes of RIRS.

## Data Availability

The original contributions presented in the study are included in the article/supplementary material. Further inquiries can be directed to the corresponding author.
